# Techniques for Detection of Clinical Used Heparins

**DOI:** 10.1155/2021/5543460

**Published:** 2021-05-06

**Authors:** Binjie Li, Huimin Zhao, Mingjia Yu

**Affiliations:** ^1^Beijing Advanced Innovation Center for Soft Matter Science and Engineering, Beijing University of Chemical Technology, Beijing, China; ^2^College of Life Science and Technology, Beijing University of Chemical Technology, Beijing, China

## Abstract

Heparins and sulfated polysaccharides have been recognized as effective clinical anticoagulants for several decades. Heparins exhibit heterogeneity depending on the sources. Meanwhile, the adverse effect in the clinical uses and the adulteration of oversulfated chondroitin sulfate (OSCS) in heparins develop additional attention to analyze the purity of heparins. This review starts with the description of the classification, anticoagulant mechanism, clinical application of heparins and focuses on the existing methods of heparin analysis and detection including traditional detection methods, as well as new methods using fluorescence or gold nanomaterials as probes. The in-depth understanding of these techniques for the analysis of heparins will lay a foundation for the further development of novel methods for the detection of heparins.

## 1. Introduction

Heparins are highly sulfated linear glycosaminoglycans, exhibiting prominent heterogeneity. Pharmaceutical heparins are generally obtained from bovine lung and porcine mucosa [1, 2]. The chemical properties of heparins derived from different sources including molecular mass distribution, pattern of sulfation, and purity will lead to discrepancies in biological activities and clinical drug safety. As one of the most widely used clinical anticoagulants [3], the relationship between curative effect and dose is not clear. Furthermore, it is the fact that the adverse effects during the clinical uses and the contamination of heparins such as oversulfated chondroitin sulfate (OSCS) [4] have become common in clinical use, which reveals that the precise methods for the detection and analysis of heparins are needed to be developed.

## 2. Classifications of Heparins

Heparin was first found by McLean and Howell at Johns Hopkins University [5] as a successful anticoagulant for over 80 years and its main chemical structure was described as a highly sulfated linear polysaccharide belonging to the family of heparan sulfate (HS) in glycosaminoglycan (GAG) [6, 7], which is composed of sulfated repeating 1⟶4-linked disaccharide units, like *β*-D-uronic acid and D-*N*-glucosamine (GlcN). Clinical used heparins can be divided into the following three categories by their molecular weight (with an average MW of 12–15 kDa and a MW range of 5–40 kDa) [8], including heparin, low-molecular-weight heparin (LMWH), and ultralow-molecular-weight heparin (ULMWH) [9, 10].

### 2.1. Heparin/Unfractionated Heparin (UH)

#### 2.1.1. Nature Product of Heparin/Unfractionated Heparin (UH)

Heparins can be extracted from many animal sources [11]. The first clinical used heparin was extracted from bovine lung and then shifted into other animal tissues, owing to the lack of bovine lung in World War II when beef had become a food source [12]. Nowadays, most pharmaceutical heparin is extracted from porcine mucosa. It has been reported that heparin derived from porcine might be more effective in anticoagulant activity than heparin derived from bovine after structural and functional analysis and less likely leading to adverse effect of bleeding [2, 13]. Due to the risk of bleeding complications in heparin derived from bovine, the detection and analysis of heparins have been hotspots of the current research in order to remove heparins derived from other animal sources as a high anticoagulant risk factor in heparin products.

#### 2.1.2. Biosynthesis of Heparin/Unfractionated Heparin (UH)

**Figure 1:**
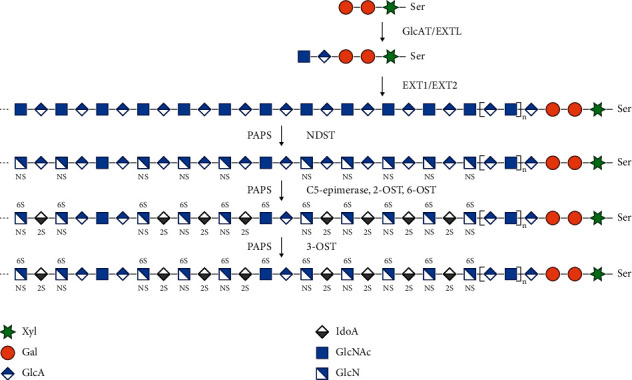
Biosynthesis of heparin/unfractionated heparin (UH). Xyl, xylose; Gal, galactose; GlcAT, glucuronosyltransferase; EXTL, initial N-acetyl glucosaminyl transferase; NDST, N-deacetylase/N-sulfotransferase isoforms; PAPS, 3'-phosphoadenosine-5'-phosphosulfate; NS, N-sulfated GlcN; 2S, 2-O-sulfated IdoA; 3S, 3-O-sulfated GlcNS; 6S, 6-O-sulfated GlcNS; *n* = 1 or 2.

The biosynthetic heparin is made from nonanimal sources [14, 15], which relies on a chemoenzymatic system. Mainly, the biosynthetic pathway of heparin has been divided into the following three parts [16]. The first part is using polysaccharide synthases to polymerize activated monosaccharides into heparosan, which is the starting polysaccharide in heparin biosynthesis with a repeating 1⟶4-linked *β*-D-glucuronic acid (GlcA) and D-*N*-acetylglucosamine (GlcNAc) [17] disaccharide unit. The second part includes the chemical transformation from an *N*-acetyl intermediate to an *N*-deacetylation and *N*-sulfonation intermediate with proper molecular weight, which is similar to commercial heparin and appropriate composition. The third part involves several groups of enzymes to modify the heparosan into HS/heparin, including C5-epimerase, 2-O-sulfotransferase (2-OST), 3-O-sulfotransferase (3-OST), and 6-O-sulfotranferase (6-OST). The most common sequence of HS/heparin is the iduronic acid (IdoA)2S (1⟶4) GlcNS6S residue, trisulfated disaccharide, while the less commonly occurring sequence is IdoA/GlcA (or very rarely a GlcA2S) 1⟶4 linked to a GlcNAc or GlcNAc6S (or rarely a GlcNS3S6S) residue (Figure 1). HS is structurally similar to heparin [18].

Although there are various advantages on biosynthesis of heparin, including extraction from nonlimited species, the product free from animal viruses' contamination and operations for better control, some problems still exist. The nonnegligible one is to match the clinical used products' chain length and structure with the standard heparins.

### 2.2. Low-Molecular-Weight Heparin (LMWH)

Low-molecular-weight heparin is a general term for heparin with an average molecular weight between 4500 and 5000 Da [19] prepared by depolymerization of UH through chemical or enzymatic pathways. Since LMWH's chains are smaller than UH's, it shows several superiorities. For instance, compared to UH, LMWH has relatively lower binding performance with thrombin, macrophages, and endothelial cells, leading to a lower ability to decrease thrombin activity but a longer half-life in plasma [20]. Meanwhile, LMWH has better bioavailability at low doses and is more predictable between dose and response characteristics. Therefore, LMWH has been widely used in clinical practice.

A variety of the clinical used LMWH [21] is prepared from UH by chemical or enzymatic ways. There are four main methods of depolymerization, including nitrous acid depolymerization (e.g., nadroparin calcium, dalteparin sodium, reviparin sodium), benzylation followed by alkaline depolymerization (e.g., enoxaparin sodium), peroxidative depolymerization (e.g., ardeparin sodium), and heparinase enzymatic depolymerization (e.g., tinzaparin sodium). Although LMWH's chains are smaller than UH's, there are many chains that are not sufficient for liver's elimination, which might give rise to a hemorrhagic tendency. Hence, the analysis of LMWH's content and concentration is indispensable.

### 2.3. Ultralow-Molecular-Weight Heparin (ULMWH)

ULMWH has even smaller chain lengths in the range of 1.5 to 3.5 kDa. Hence, ULMWH has a lower bleeding risk, longer plasma half-life, and better bioavailability as well as the ability to penetrate the blood-brain barrier. Clinical commonly used ULMWH such as fondaparinux sodium is usually chemically synthesized through one-pot synthesis [22]. Chemical synthesis can make the product purer and its structure more predictable, whereas by-products still exist so that the amounts of them are required to be quantified before launched on the market or clinical used.

## 3. Anticoagulant Mechanism of Heparins

Blood freely circulates in healthy individuals' arteries and veins. Nevertheless, as soon as the hemostatic system is triggered, the biochemical cascade reactions leading to the hemostatic effects will take place with the aggregation of platelets and fibrin. Heparins have been regarded as effective anticoagulants for several decades. This part mainly elaborates the anticoagulant mechanism of heparins.

### 3.1. The Process of Hemostasis

**Figure 2:**
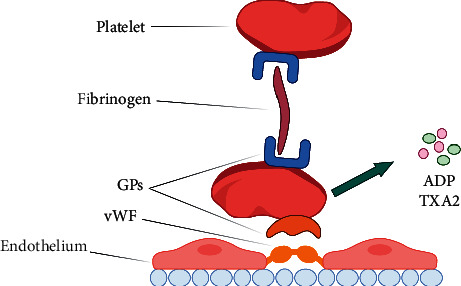
The primary process of hemostasis. The adhesion and aggregation of mediated by von Willebrand factor (vWF) and glycoproteins (GPs), and the release of ADP and TXA2.

Primary process of hemostasis is defined as the platelet plug's formation after blood vessel's damage. The platelet aggregation is mainly mediated by fibrinogen which binds to the platelet surface receptors, principally including glycoprotein (GP) receptor Ib. Furthermore, the connection between the platelet surface receptors and Von Willebrand factor (vWF) [23] on the endothelial collagen has put platelet and endothelium together, leading to the platelet adhesion. Meanwhile, the activated platelet's degranulation releases different kinds of factors including adenosine diphosphate (ADP), thromboxane A2 (TXA2) [24], and serotonin [25, 26] (Figure 2) that might have different impact on platelet including the platelet aggregation as well as vasoconstriction.

**Figure 3:**
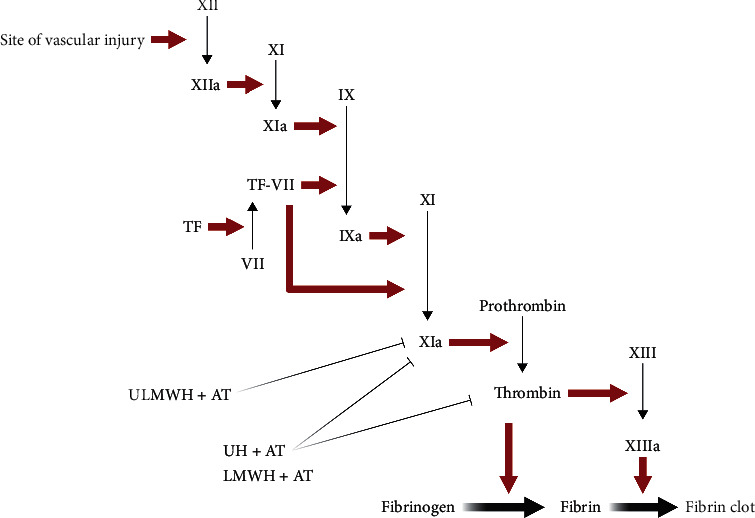
Main coagulation cascade reactions activated by vascular injury and heparins' anticoagulation.

Secondary process of hemostasis is defined as the insoluble fibrin's formation mediated by biochemical cascade reactions (Figure 2). There are two pathways in secondary hemostasis cascade reactions including the intrinsic and extrinsic pathways. The intrinsic pathway is triggered by coagulation factors XII and XI. Factor XII's local concentration increases when factor XII is attached to the damaged vessels' negatively charged surface where phospholipids exposed. Then, factor XII is activated to become factor XIIa by endodermal collagen. The extrinsic pathway, also called tissue factor pathway, is the prior in plasma-mediated hemostasis. The membrane protein tissue factor (TF) contacts with factor VII in plasma and then triggers the extrinsic pathway leading to the formation of TF-VIIa complex. TF-VIIa complex and factor XIIa coactivate and catalyze the transformation of factor from XI to IXa, while the TF-VIIa complex and factor IXa catalyze the conversion of factor X to factor Xa. Then, factor Xa catalyzes the formation of prothrombin (factor II) to thrombin (factor IIa). As the final serine protease cascade reactions starts, thrombin has multiple functions in coagulation including activating platelets, factors V, VIII, IX, protein C, and fibrinolysis inhibitor in order to promote the coagulation cascade reactions as well as convert fibrinogen into fibrin. The last step in the clotting process is the conversion from fibrin to insoluble fibrin clot, which is mediated by factor XIIIa [20] (Figure 3).

### 3.2. Heparins' Anticoagulation

Antithrombin (AT) is a glycoprotein and a serine protease inhibitor produced by the liver, which can inhibit various activated proteases associated with coagulation, including TF-VIIa complex [27], factor IXa [28], factor Xa [29], and thrombin by covalently binding to their serine residues. Moreover, together with heparins, AT plays a key role in the inhibition of serine protease by forming a thrombin-AT-heparin ternary complex with the presence of thrombin.

UH is commonly used in clinic which is extracted from animal tissues, mainly from porcine intestinal mucosa with the average molecular weight from 10 to 20 kDa. UH's anticoagulation activity is mainly determined by its unique pentasaccharide sequence which gives it high affinity to bind with AT, whereas only about one-third of UH has the unique structure that could bind to AT, leading to only one-third of UH administered to patients having anticoagulant activity mediated by AT. UH accelerates the inhibition of factor Xa by endogenous AT. The close integration between UH and AT/thrombin gives rise to a more significant inhibition of thrombin activity. The clinical used LMWH is mostly prepared by UH's depolymerization containing the pentasaccharide sequences like UH, which leads to the same anticoagulant mechanism as UH. However, the heparins contain less than 18 saccharides making it unable to bind thrombin [30]. Above all, compared with UH and LMWH, ULMWH exerts effects by catalyzing the AT-mediated inhibition of factor Xa's activity. In addition to the combination with AT, heparins bind to heparin cofactor II and platelets leading to the inactivation of factor IIa [19].

## 4. Heparins' Therapeutic Applications

Heparins are suitable for many clinical indications, and the therapeutic applications can be mainly divided into the following two categories: anticoagulant [11] and anti-inflammatory.

### 4.1. Heparins' Therapeutic Application as Anticoagulants

Heparins' major clinical application is for the treatment of venous thromboembolism (VTE) including pulmonary embolism (PE) [31, 32] and deep venous thrombosis (DVT). UH has been the standard medication for the treatment of VTE for more than half a century, whereas it had been demonstrated that LMWH' dose-determined treatment was as safe and efficient [33] as UH's dose-adjusted treatment for PE [34] and DVT [35]. Hence, LMWH is the preferred drug of initial therapy in VTE. Dose-determined LMWH administration for once or twice daily shows the convenience that pharmacokinetic characteristics are more predictable without the routine laboratory monitoring [33], whereas there are several situations that UH may be more suitable, such as when patients are at high risk of bleeding or with impaired renal function. When using UH, the anticoagulant effect can be terminated more quickly as soon as the infusion is stopped and if necessary, protamine's reverse effect on coagulation is more significant in patients at risk for bleeding. Moreover, LMWH's drug clearance tends to be reduced, which would make LMWH prone to bleeding, especially in impaired-liver-function patients [12, 36]. During the treatment of VTE, UH or LMWH is given via intravenous or subcutaneous injection. Depending on the severity of VTE, the therapeutic approach and dose would be different. For example, the initial treatment for patients at moderate risk of PE [37] requires intravenous or subcutaneous UH, or subcutaneous LMWH for the first 5 to 10 days, a dosage varying from 300 IU/kg for UH or 170–200 IU/kg for LMWH, respectively.

There are special cases in patients with VTE, who have pregnancy or suffer from cancer. Compared with nonpregnant women, the risk of VTE increases approximately fivefold in pregnant women and the risk remains increased until about 12 weeks postpartum, leading to about 10% maternal deaths in developed countries [38]. Both UH and LMWH are safe for the treatment and prophylaxis of VTE [39] in patients with pregnancy, because they do not pass the blood brain barrier (BBB), whereas the use of vitamin K antagonists (VKAs) capable of crossing the BBB will cause teratogenicity in the first trimester and intracranial hemorrhage in the third trimester in infants. Furthermore, compared with UHs, LMWHs are the preferred agents and could be injected subcutaneously twice a day, for they are less likely to cause the side effects such as thrombocytopenia or osteoporosis [20, 40].

The risk of VTE increases approximately fourfold in patients with cancer when compared with other patients. Furthermore, the mechanism of antitumor property in heparins is mainly due to the inhibition in the formation of blood vessels around tumors, since the growth of tumor larger than 1 mm^3^ is dependent on angiogenesis [20]. LMWH is the primary therapeutic agents of VTE therapy for cancer patients, including the treatment of catheter-related thrombus [40]. The initial duration of treatment with LMWH will last for 3–6 months, and the treatment could be continued indefinitely if the cancer is not cured for cancer patients.

### 4.2. Heparins' Therapeutic Application as Anti-Inflammatory Agents

Heparins can be administered intranasally besides intravenous and subcutaneous medication, which could work as anti-inflammation drugs. Heparins could inhibit the release of inflammatory cell-derived mediators and the recruitment of inflammatory cells to tissues by modulating the interaction between leukocytes and vascular endothelial cells, leading to the inhibition of nasal airway pressure [12], leukocyte infiltration, eosinophilic cell migration, and eosinophilic cationic protein. Hence, it has been demonstrated that the intranasal administration of heparin might be used in the treatment of allergic rhinitis and asthma [20].

Despite the widespread application of heparins, several adverse reactions still exist, such as hemorrhage, heparin-induced thrombocytopenia [41], osteoporosis [42], elevation of transaminases, and hypersensitivity [20]. Hence, the clinical analysis and trials of heparins are indispensable.

## 5. Analysis of Heparins

### 5.1. Preclinical Samples Analysis

**Figure 4:**
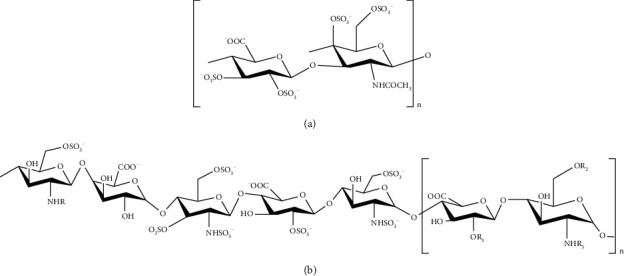
(a) The repeat disaccharide components of OSCS. (b) The sequence of pentasaccharide binds with antithrombin III and repeat disaccharide components of heparin (*R*_1_ = H SO_3_^−^; *R*_2_ = SO_3_^−^; *R*_3_ = H SO_3_^−^, acetyl).

Heparins have been widely used as anticoagulant drugs, whereas heparins are extracted or synthesized from different sources, with different molecular weight. And there are heparins-induced adverse reactions existing during the clinical treatment. Furthermore, the adulteration of OSCS (Figure 4(a)) in heparin (Figure 4(b)) gives rise to anaphylactic shock, resulting in a global crisis [4]. Hence, the detection and quantification of heparins before being applied to clinical practice is indispensable. There are several methods for the analysis of heparins, including single technique and multiple combinations of techniques for detection and analysis (Table 1).

#### 5.1.1. Single Techniques for Detection and Analysis of Heparins

*(1). Nuclear Magnetic Resonance (NMR)*. NMR is a physical process in which the spin level of a nucleus with a nonzero magnetic moment shows Zeeman splitting under the action of an external magnetic field, and a certain radio frequency of saccharide is absorbed by resonance. NMR spectroscopy has been recognized as a powerful analytical method for over 60 years. NMR spectral parameters such as the chemical shift, the J-coupling, nuclear Overhauser effects, and residual dipolar couplings convert into angular or distance data and then provide conformation at the atomic level [52]. In heparins' analysis, samples are prepared by dissolving in D_2_O and lyophilizing to remove exchangeable hydrogen from the backbone of the structure. Then, the samples are redissolved in D_2_O to minimize the HDO peak in the NMR spectrum [49, 50]. Through one-dimensional (1D) ^1^H-NMR spectra and two-dimensional (2D) heteronuclear single quantum coherence spectroscopy- (HSQC-) NMR, St Ange et al. [50] verified the structural similarities and differences between bovine and porcine heparins. By the method of NMR, it reveals that fact of the glucosamine substitution and chiral properties of uronic acid residues in heparin and LMWH [53].

*(2). Mass Spectrometry.* In the process of mass spectrometry, each component of the samples is ionized in the ion source to generate charged ions with different ratios of charge to mass, and the ion beam is formed under the action of the accelerating electric field and then entering the mass analyzer [54]. In the mass analyzer, electric and magnetic fields are used to generate the opposite velocity dispersion, and they are, respectively, focused to obtain the mass spectrum, so as to determine the molecular weight of the components. Ola et al. [55] developed a method which combined electrospray ionization mass spectrometry (ESI-MS) and tandem mass spectrometry (MS^n^). In the study, heparins were depolymerized by heparin lyases, and the mixture was compatible with ESI-MS and MS^n^ without any further purification. Through the analysis of the substance of mixture and standard by using the combination of ESI-MS and MS^n^ techniques, it obtained the composition analysis and quantification of eight commercially available disaccharides derived from bovine and porcine heparins.

*(3). Electrophoresis.* Electrophoresis is a technique in which charged particles are separated by moving at different speeds in an electric field. Since the adulteration of heparins with OSCS, methods for heparins purity analysis have been developed rapidly [4], including capillary electrophoresis (CE) and polyacrylamide gel electrophoresis [12]. The mechanism of CE separation is based on the ratio of molecular charge to volume which is particularly effective for the analysis of polyanions such as heparin and OSCS [45]. Somsen et al. [56] replaced sodium ions with Tris in the high concentration buffer, realizing the effective separation of heparin and OSCS. CE is also an effective method to characterize heparin-bovine serum albumin (BSA) interaction, which has been carried out by microfluidic chip [57]. PAGE uses polyacrylamide gel as the supporting medium which has a network structure, and molecular sieve effect. Hence, PAGE becomes an efficient method to separate heparins and their impurity, mainly OSCS, by exploiting the different properties in their molecular sizes, conformations, and charges [45].

*(4). High-Performance Liquid Chromatography (HPLC).* HPLC takes liquid as the mobile phase and uses a high-pressure infusion system to pump single solvent with different polarity or mixed solvents and buffers or other mobile phases into a column equipped with a chromatographic column. After each component in the column is separated, it enters the detector for inspection, realizing the analysis of sample. There are two main types of HPLC which are involved in the analysis of heparins including strong anion-exchange (SAX)-HPLC and weak anion-exchange (WAX)-HPLC. In SAX-HPLC, because of the reversible ionic interaction between a charged analyte and oppositely charged stationary phase, the analyte with low charge and small size will be eluted first. In heparins' analysis, highly negatively charged GAG is preserved on anion-exchange columns and then eluted with gradient inorganic salt solution with high ionic strength [47]. Miller et al. [58] developed a SAX method using volatile salt buffer in the process of UH analysis, realizing the purification of heparin oligosaccharides and the subsequent MS analysis. The mechanism of WAX-HPLC is similar to that of SAX-HPLC; the main difference is that the interaction between the analyte and the stationary phase is relatively weaker. Hashii et al. [59] isolated heparins and OSCS from heparin sodium and heparin calcium by WAX.

*(5). Size Exclusion Chromatography (SEC).* SEC is a chromatography technique in which molecules are separated according to the size of the samples. In the field of heparins' analysis, Ouyang et al. [48] used multiple angle laser scattering (MALS) and refractive index (RI) in SEC to detect the difference in light scattering intensity between the background and the chromatographic analytes, which is vital to calculate the accurate molecular weight of UH and LMWH. Gel permeation chromatography (GPC) also belongs to the method of SEC, which uses organic solvents as the mobile phase passing the measured polymer solution through a column with different pore sizes to measure the molecular weight of heparins [50].

#### 5.1.2. Multiple Combinations of Techniques for Detection and Analysis of Heparins

*(1). Liquid Chromatography- (LS-) MS.* LS-MS is a combination of LS and MS, LS can effectively separate the organic compounds in the samples, and MS is able to analyze the separated organic compounds in order to obtain the information on their molecular weight, structures, and concentrations. It is worth noting that, before the LS-MS heparin analysis, the samples need to be boiled and centrifuged to remove the heparinase; then the supernatants required to be lyophilized and redissolved [49, 53]. Ouyang et al. [48] developed LC-MC analysis method which can detect adulterant of 10% bovine or ovine heparin in porcine heparin.

*(2). HPLC-Ultraviolet Spectroscopy.* HPLC is combined with spectroscopy in the form of the UV detector, turning into a powerful detection technique [60]. The mechanism of UV detector is to absorb ultraviolet light to determine the individual quantities of each component in the samples. The amount of UV light absorbed by each component is measured by passing UV light through the eluted samples. St Ange et al. [50] developed a method combined with HPLC-UV spectroscopy to analyze the disaccharide composition after the depolymerization of heparins derived from bovine and porcine.

*(3). CE-MS.* CE is a technique of electrophoresis based on the separated differences of components in mobility and distribution behavior. MS is a technique for quantitative and structural analysis by measuring the mass and strength of the ions in the samples. Combined techniques CE-MS has shown high efficiency and sensitivity for the analysis of heparin oligosaccharides and LMWH [61, 62]. Ouyang et al. [49] developed a CE-MS method, in which the jobs involved with the removal of sulfonic group and the selective chemical derivation of oligosaccharides are not required, compared to LS-MS. Furthermore, the oligosaccharides of heparin have a large negative charge, which are ideal preconditions for tandem mass spectrometry analysis [51]. Researches have also shown that, by using a novel electrokinetic CE-MS interface, the analysis of GAG oligosaccharides from heparin disaccharide to LMWH efficiency has been greatly improved, leading to the generation of high sensitivity and throughput analysis for heparins [61].

#### 5.1.3. New Techniques for Detection and Analysis of Heparins

*(1). Fluorescent Probe.* Fluorescence detection of biomolecules has attracted great interest because of its high sensitivity [63], high selectivity [64], high throughput [65], and low cost [66]. Short peptides composed of less than three to four amino acid residues can self-assemble in aqueous solution to form definite nanostructures. Lee et al. [67] synthesized a heparin-induced self-assembly fluorescent peptide probe with an aggregation-induced emission fluorophore, which was able to form nanoscale aggregates binding with heparins in order to measure the amount of heparins. Since OSCS has an inhibitory effect on heparinase, Mehta et al. [68] had developed a method which was able to detect as low as 0.0001% of OSCS contamination in heparins by adding heparinase into the mixture of heparins and detected the fluorescent change. If heparins were contaminated by OSCS, the changes in fluorescence intensity were reduced compared to the uncontaminated standard.

Cheng et al. [69] developed a composite which was prepared from graphite carbon nitride quantum dots, and silver nanoparticles. The fluorescence of the composite was strongly decreased under the coating of polyethylenimine (PEI), whereas PEI had a higher affinity for heparins than the composite, so the fluorescence of the system was enhanced after adding heparins. Moreover, the composite was also applied to the fluorometric determination of heparins, such as the detection of heparin in human serum samples.

There are also other types of visible fluorescence immunoassay impregnated on paper that may have great potential for heparin detection. Huang et al. [70] developed a paper-based analytical device (PAD) that Ag^+^ fluorescently quenched CdTe quantum dots (QD) on nitrocellulose films by distance dependent silver ion exchange reaction, followed by Sandwich immune reactions between silver nanoparticles labelled secondary antibodies and the microtitration pores coated with primary antibodies to test human serum samples. Meanwhile, Lv et al. [71] produced wet NH_3_ by sandwich immunoassay using highly functionalized gold nanoparticles with glutamate dehydrogenase and secondary antibody. The wet NH_3_ could trigger the structural change of the paper impregnation analysis device coated with NH2-MIL-125 (Ti), which showed good visible fluorescence intensity. Moreover, Qiu et al. [72] developed an integrated PAD that combined DNA-gated mesoporous silica nanocontainers with biological controlled release systems for the visual fluorescence detection of CdTe/CdSe QD-enzyme impregnated paper.

*(2). Gold Nanomaterial Labelled Sensors.* There are mainly two kinds of gold nanomaterials for the detection of heparins, including gold nanoparticles and gold nanorods. Gold nanomaterials have unique optical properties and their light scattering is clearly stronger than that the emission organic dyes [73].

Fu et al. [74] developed an ultrasensitive detection method based on the self-assembly of gold nanoparticles (AuNPs) on the surface of graphene oxide (GO) with the polycationic protamine as medium. The electrostatic interaction between protamine and gold nanoparticles induced the self-assembly of gold nanoparticles (AuNPs) on the surface of GO accompanied by AuNPs turning into blue. Due to the strong affinity with protamine, heparins with polyanion were interfered with the self-assembly of AuNPs. Hence, with the increase of heparins concentration, the number of AuNPs self-assembly decreased and the color of AuNPs color changed from blue to red. Wang et al. [73] developed the plasmonic gold nanorods (AuNRs) as light scattering probes to detect the concentration of heparins. In the reaction, the Au atoms in AuNRs were oxidized by Fe^3+^ to Au^+^, which then formed AuTu_2_ with thiourea. Then, the reaction was promoted by heparins to form a complex with AuTu_2_ due to the strong electrostatic interaction. During this process, the light of AuNRs scattered from red to green. And the time taken for light scattering depended on the concentration of heparins.

AuNPs are able to greatly improve the sensitivity of detection by interacting with other materials, which can be used for reference to the future application of heparin detection.

Tang Group [75] developed a label-free adapter sensor based on the change in color/absorbance induced by salt-induced aggregation of AuNPs in order to detect the samples of adenosine 5'-triphosphate (ATP). After the addition of the target ATP, the aptamer-based hairpin probe would expose a new sticky end for the strand-displacement reaction with another complementary hairpin leading to the decreasing single-stranded DNA and the formation of double-stranded DNA, which would release more AuNPs, triggering a red-to-blue color change by salt-induced aggregation of AuNPs. The sensor with high sensitivity was able to detect the concentration of ATP as low as 1.0 nM. Furthermore, this technique could be applied to the quantitative detection of other biomolecules by changing the sequence of the corresponding aptamer.

Clinical drug detection is mainly aiming at the quantitative analysis of drug content in serum. Cai et al. [76] developed a sensor that could detect sensitive photoelectrochemical (PEC) aptasensing from human serum samples by exciton plasma interaction between graphene nanosheets (GN) coated with AuNPs and CdS QDs. This sensor has good reproducibility and high sensitivity with the limit of detection of antigen in 0.52 pg/mL.

Zhuang et al. [77] developed a PEC sensing platform to detect the T4 polynucleotide kinase activity based on AuNPs decorated g-C_3_N_4_ nanosheets after the deoxyribozyme-mediated catalytic precipitation amplification. Meanwhile, due to the introduction of AuNPs on the surface of g-C_3_N_4_, the surface plasmon resonance enhanced the light collection and the separation of the photo-generated e^−^/h^+^ pairs leading to its highly sensitive activity detection. Furthermore, Lv et al. [78] developed the sensor which was assembled on the 3D printing device based on the rolling ring amplification. By using D-A F8BT/g-C_3_N_4_ type II heterojunction nanocomposite as the photoelectrode material, this sensor improved the photocurrent effect of g-C_3_N_4_, enhanced the built-in electric field, and promoted the generation of electron holes with the local plasma resonance of AuNPs, which further improved the photoelectric effect leading to a highly sensitive detection.

### 5.2. Clinical Sample Analysis of Heparins

The usual samples for clinical heparins' analysis are patients' plasma or serum. The measurable indicators mainly include D-dimer and activated partial thromboplastin time (APTT). D-dimer is a reliable and sensitive indicator of fibrin deposition and stabilization, which is also a sign of thrombosis. The concentration of D-dimer measured by enzyme linked immunosorbent assay (ELISA) can be used to semiquantitatively analyze heparins in plasma, whereas D-dimer also presents a high concentration in certain conditions unrelated to thrombosis, leading to a false positive result without symptoms of thrombosis, which might give an inaccurate analysis of heparins [79]. The APTT assay is performed on platelet poor plasma (PPP) and then heated to 37°C by adding to preheated phospholipid and a contact activator. Subsequently, the mixture was incubated for 3–5 minutes to activate the contact pathway; then, calcium was added to begin clotting. APTT is the time from adding calcium to the formation of fibrin clots. Furthermore, there is a good correlation between APTT and heparin dose; hence, APTT can roughly define the plasma heparin level [30].

Furthermore, there are new detection methods applied in clinic. Jang et al. [80] developed the self-assembled conjugated fluorescent micelles which amplified the fluorescence response to heparins and had been applied to the highly sensitive detection of heparins in serum. Meanwhile, AuNPs with the same principle as the above gold nanomaterials had been applied in the clinical detection of heparins. Yuan et al. [81] developed a high sensitivity SPR sensor to detect heparins in serum in a portable optical fiber SPR sensing system. Hence, the sensor is expected to be a practical tool for real-time monitoring of heparins.

## 6. Conclusions

There are many effective methods for clinical used heparins' analysis, including both single techniques: NMR, MS, CE, PAGE, HPLC, and SEC and multiple combinations of techniques: LS-MS, HPLC-UV, and CE-MS, which mainly detect the D-dimer concentration and APTT in the analysis of clinical samples. Despite the fact that several new technologies including fluorescent probe and gold nanomaterial labelled sensors have been developed to accurately detect heparin, there is still plenty of room to investigate more high-sensitive, high-throughput, and rapid clinical methods for analysis of heparins. Furthermore, heparins have not only been shown to have powerful therapeutic effects in coagulation related diseases, but also been regarded as a therapy for COVID-19 through antiviral and anti-inflammatory effects [82, 83]. Hence, component analysis of heparin in patients' body fluids and comprehensive efficacy assessment are the top priority for follow-up work.

## Figures and Tables

**Table 1:** The characteristics of routine heparin analysis and detection methods.

Method	Samples	Purpose	Loading quantity	LOD/LOQ	Ref nr
NMR	Standards	To quantify the degree of porcine derived heparin adulteration with bovine derived heparin	70 mg/mL	2% w/w	[43]
MS	Tissue/standards	To identify the disaccharide components retaining the differential isomerization configuration of the parental heparin chain	—	—	[44]
CE	Standards	To quantify the degree of heparin adulteration with OSCS	5-10 mg/mL	0.05% w/w	[45]
PAGE	Tissue/standards	To quantify the degree of heparin adulteration with OSCS	0.16-4 mg/mL	0.1–5% w/w	[45]
SAX-HPLC	Standards	To analyze the structure and content of oligosaccharides after digesting heparin by heparinases	2.5 mg/mL	LOD 0.1% LOQ 0.3%	[46]
WAX-HPLC	Standards	To isolate and quantify the degree of heparin adulteration with OSCS	1 mg/mL	0.025–0.075% w/w	[47]
SEC	Standards	To quantitatively analyze the molecular weight of UH and LMWH	0.02 mg/mL	—	[48]
LC-MS	Standards	To detect heparin mixture derived from different sources by analyzing the composition difference of disaccharide and tetrasaccharide	Disaccharide: 1 mg/mL. Tetrasaccharide: 2 mg/mL	—	[49]
HPLC-UV	Tissue/standards	To analyze the disaccharide and tetrasaccharide composition of heparin oligosaccharides	Disaccharide: 250 *μ*g/mL tetrasaccharide: 5 mg/mL	—	[50]
CE-MS	Standards	To analyze the disaccharide components of heparin oligosaccharides	100 *μ*g/mL	—	[51]

## Data Availability

No data were used to support this study.
